# COVID-19 Outbreak Response for an Emergency Department Using In Situ Simulation

**DOI:** 10.7759/cureus.7876

**Published:** 2020-04-28

**Authors:** Marcus Jee, Daniel Khamoudes, Aoife M Brennan, John O'Donnell

**Affiliations:** 1 Emergency Department, Galway University Hospital, Galway, IRL; 2 Emergency Medicine, Galway University Hospital, Galway, IRL; 3 Anaesthesiology, Galway University Hospital, Galway, IRL; 4 Emergency Department, University College Hospital Galway, Galway, IRL

**Keywords:** covid 19, emergency medicine, emergency department, medical simulation, in situ simulation, system testing, latent safety hazards, irish emergency department, anaesthesiology, outbreak response

## Abstract

In January 2020, the WHO declared COVID-19 an epidemic in China and the possibility of evolving into a pandemic. During early phases, most emergency departments did not have contingency plans for an outbreak of this scale and therefore necessitating adequate preparation.

This study aims to describe the utility of in situ simulation in identifying system errors and latent safety hazards in response to preparation for the expected COVID-19 surge. We also aim to describe the corrective measures taken to improve our outbreak response locally. We hope that others may be able to use this information as foresight in preparing their own departments for this outbreak.

The emergency department and anesthesiology department of Galway University Hospital conducted a series of multidisciplinary, in situ simulations to rapidly identify operational errors and latent safety hazards in response to this outbreak. Each simulation involved an interdisciplinary response to a suspected/COVID-19 patient. The cases were used as a training opportunity for staff and ultimately a platform to expeditiously implement system changes in response to deficits identified during the simulations.

Conclusively, with the complexities and intricate structure of every emergency department, we understood that preparation for an outbreak requires evaluation of the current system before implementing any changes. It is not a "one size fits all" concept. Therefore, conducting in situ simulations and the use of foresight, is pivotal as it could prevent loss of resources and time in preparing for an outbreak.

## Introduction

Late 2019, COVID-19 originated from the city of Wuhan, Hubei in China [1]. It was recognized as an epidemic but with the potential to be a pandemic. Subsequently, in March 2020, the World Health Organisation (WHO) declared COVID-19 a pandemic [2].

Current evidence suggests that COVID-19 is transmitted through contact, respiratory droplets, and possible fecal-oral route [3]. This, however, implies that through procedures such as tracheal intubation and other aerosol-generating procedures, COVID-19 could pose a risk to healthcare personnel [4].

As of April 9, 2020, there are 1.4 million confirmed cases, and 81,058 confirmed deaths reported on the WHO live “situation dashboard” [5]. During the time of writing, the Health Service Executive (Ireland) has already made moves to prepare for the outbreak. However, every emergency department (ED) has prepared differently due to factors such as size, level of hospital, number of cubicles, and resuscitation bays availability among many.

In situ simulation has been proven to be highly effective in identifying system failures and improving safety in acute clinical settings, such as an ED or Intensive Care Unit [6-8]. We, therefore, aimed to utilise a series of in situ simulations to identify system errors and latent safety hazards in managing an acutely unwell COVID-19 patient within our departments.

The objective of this article is to report on our system errors and latent hazards then describe our methods and corrective measures that we took to manage this outbreak. All these in hopes that others may reproduce and use it as foresight in preparation for their ED.

## Materials and methods

The ED and the Anaesthesiology Department of Galway University Hospital (GUH) conducted a series of multidisciplinary, in situ simulations to evaluate for operational errors, identify latent hazards, training and to rapidly implement change as an outbreak response to decrease viral transmission amongst staff and ensuring the safety of other patients in preparation for an outbreak.

This project was discussed with the local research, development, and quality improvement team who stated that no ethical approval needed as there was no patient involvement. This project was approved by the Emergency Medicine Clinical Lead and the Anaesthesiology Clinical Lead.

From the 4th of March to the 25th of March 2020, a series of in situ simulations were conducted in the ED and the ICU. The simulations were conducted by an ED consultant and an ED trainee currently undertaking a simulation master program, and an Anaesthetics Specialist Registrar. The scenarios were conducted during work hours on both weekdays and weekends.

We designed two scenarios for different purposes: The first, involved a walk-in, suspected COVID positive patient who subsequently deteriorated. This scenario was designed to test: the preparedness of the ED in receiving such a patient along with its precautions in place (from reception to ICU) and the second scenario, involving an acutely unwell COVID positive patient who presented via ambulance requiring immediate resuscitation. This case tested the suitability of the resuscitation room, equipment, and multidisciplinary teamwork for the transfer of a COVID positive patient.

During these scenarios, video recording was undertaken. Verbal consent was obtained from all anesthetics and ED personnel beforehand.

After every scenario was conducted, a debrief was held. This debriefing session was led by an ED consultant along with an anaesthetics consultant/SPR, along with a simulation fellow. During the debriefing, the participant’s opinion was noted. This included systems in place, hazards, equipment, staffing issues, communication, personal protective equipment issues, and latent threats. Further discussions were then held from the facilitator and observer perspective. During these discussions, systems testing and suggestions for improvements were our primary focus.

Data on the following were collected: Time from ED resuscitation room to ICU, activation time for a newly configured “Code-Blue: COVID - Anaesthetics”, security activation and the number of personnel needed. All other analysis was via observation and review.

After the initial debrief, the videos were analysed further then presented in a weekly COVID Task Force meeting aiming to solve additional issues found.

Equipment used:

1. one manikin: Simman ALS (In a resource compromised setting any manikin can be used) and clinical equipment in ED

Staff involved:

1. One ED doctor along with two nurses comprised the initial team with further reserve staff available as needed for both the sim and shop floor assessment. 2. One SIM coordinator, one facilitator, one simulation observer/fellow. 3. External/nonclinical representative from management, i.e., General Manager.

## Results

We ran a total of four simulations. A multidisciplinary team of 39 personnel took part in the simulations (Table 1).

**Table 1:** Personnel included in simulations.

Hospital general manager	1
Hospital assistant general manager	1
Assistant directors of nursing	2
ED consultants	5
Anesthetics consultants	4
Senior anesthesiologist	6
Senior ED doctors	4
Junior ED doctors	4
Nurses	8
Infection control personnel	2
Security personnel	2

We identified a number of latent safety hazards during the course of the simulations. These ranged from narrow hallways, unsuitable clinical spaces, stock issues, and a lack of available protocols to manage a pandemic. While participants deemed teamwork a key element of "what went right", we noted the involvement of too many staff in each case. This, again, was a result of a lack of protocols with no defined clear role allocations. We will detail the above findings and changes implemented in the paragraphs below.

**Figure 1:**
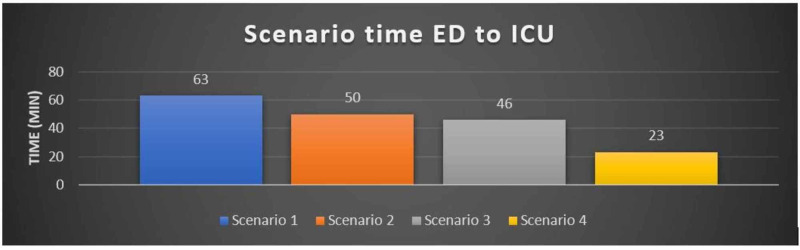
Scenario time from ED resuscitation room to ICU.

The time of scenario from ED resus to ICU recorded a mean of 45.5 minutes and a median of 48. Throughout the simulations, a steady improvement was seen in the transfer times. A significant improvement of 40 minutes (37.09%) was seen between the first and last simulation (Figure 1). These improvements were a result of continuous corrective measures based on the analysis of the previous simulation.

The CODE Blue: Anaesthetics call activation response time averaged at 3 minutes for three scenarios.

Security team activation response time averaged at 7 minutes for three scenarios.

Identified issues and implemented changes

Clinical

**Figure 2:**
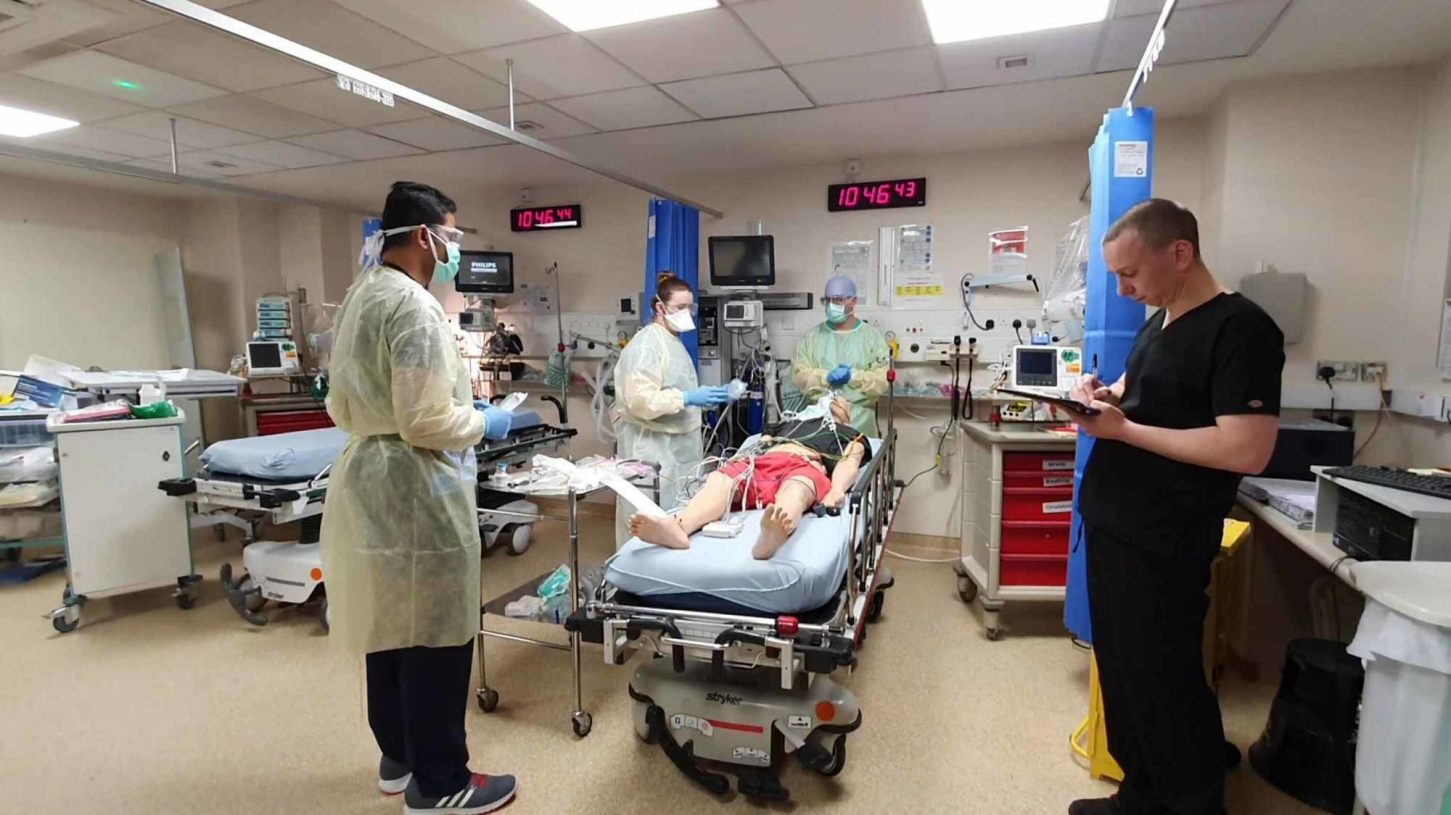
Training using in situ simulation. Facilitator (in black) seen taking notes during the simulation.

**Figure 3:**
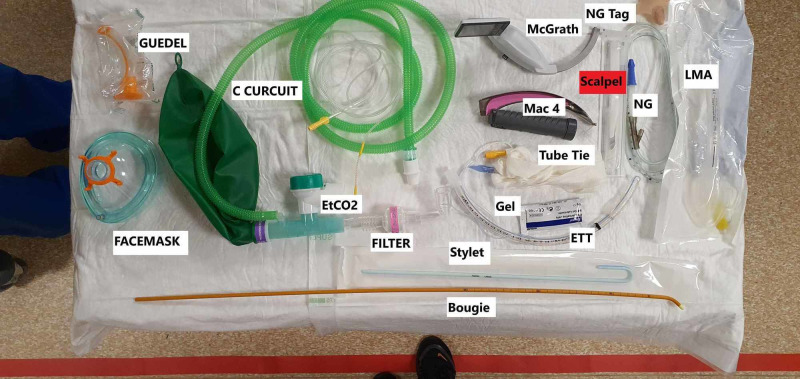
New airway trolley with only essential equipment.

1. Training was conducted via multidisciplinary in situ simulations. During these simulations, a facilitator and simulation fellow were tasked to observe and note for system failures and latent safety hazards. Additionally, all simulations were video recorded, then edited with a voice over to be distributed to the relevant personnel for educational purposes. This aimed to ensure consistency across the board in the management of any COVID-19 resuscitation case (Figure 2).

2. We found that Intubating a patient using standard procedures and equipment poses the risk of aerosolising potential COVID-19 particles and droplets. This was seen during the first simulation, where the intubator proceeded with a standard rapid sequence intubation (RSI) checklist without the use of a viral filter. We updated the existing RSI checklist and set-up a new airway trolley with only the essential equipment after receiving insights from anaesthetics clinical leads (Figure 3).

Stock

During the simulations, we found that we were running out of available personal protective equipment (PPE), i.e., filtering facepiece particles (FFP3) masks, face shields, and goggles. Upon investigation, we discovered that all PPE was kept in a single, general trolley and was only restocked once a day. This was leading to deficits in PPE when required urgently for acute cases. We, therefore, created a designated "resuscitation only" PPE trolley to be kept separately to general PPE supplies.

**Figure 4:**
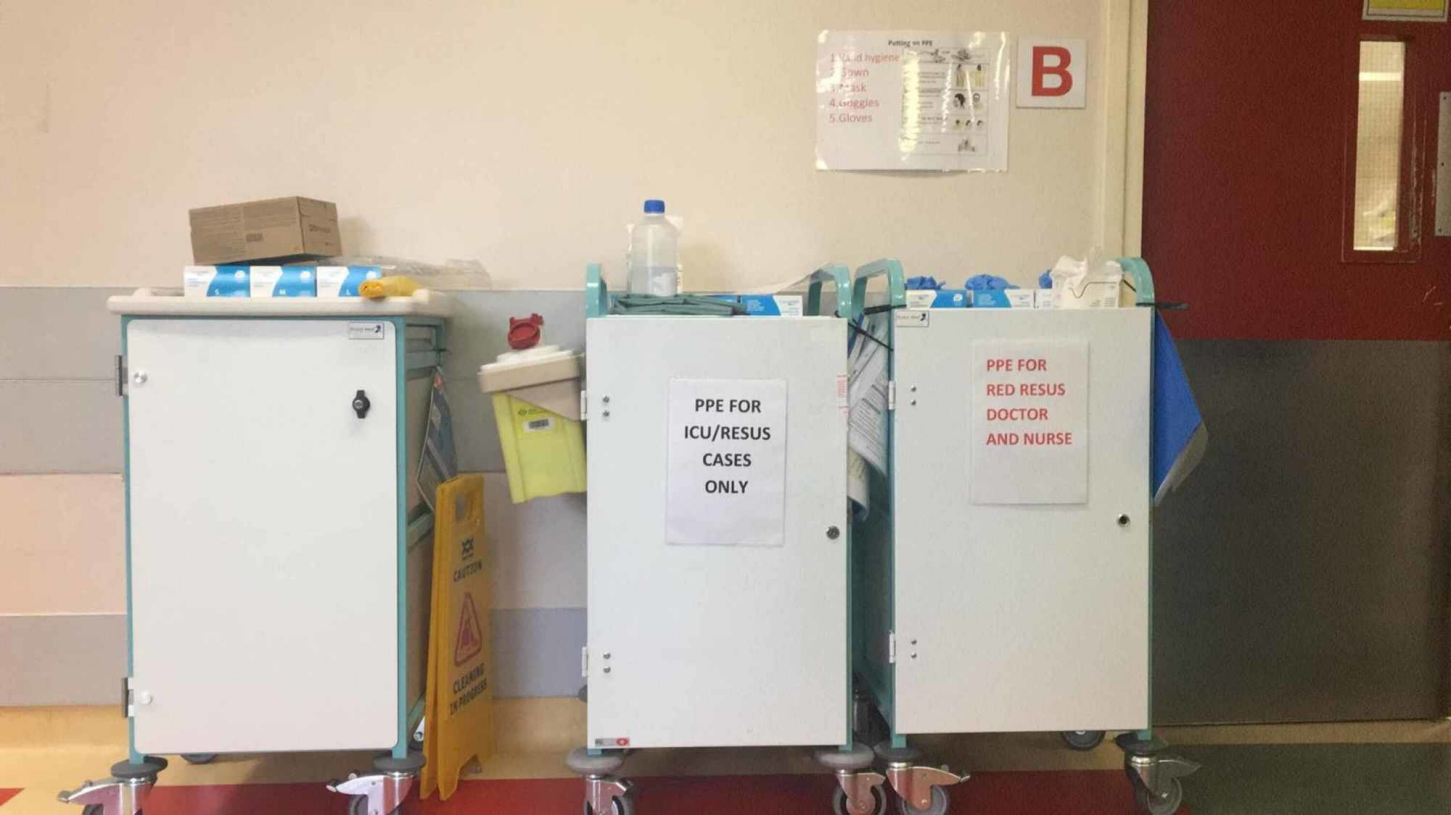
Specified PPE for resuscitation room use only. PPE: Personal protective equipment

1. A specific “resuscitation only PPE trolley” was placed in resus (Figure 4).

Logistical

We found that during transfer, the hallways were not secure. Our resuscitation room is located on the ground floor of our hospital, while the ICU is on the 3rd. This required a significant transit time through "non-covid" areas, including waiting rooms, hallways, and lifts. This resulted in significant potential to expose both public and other staff on transferring COVID-19 patients from one area to the other.

**Figure 5:**
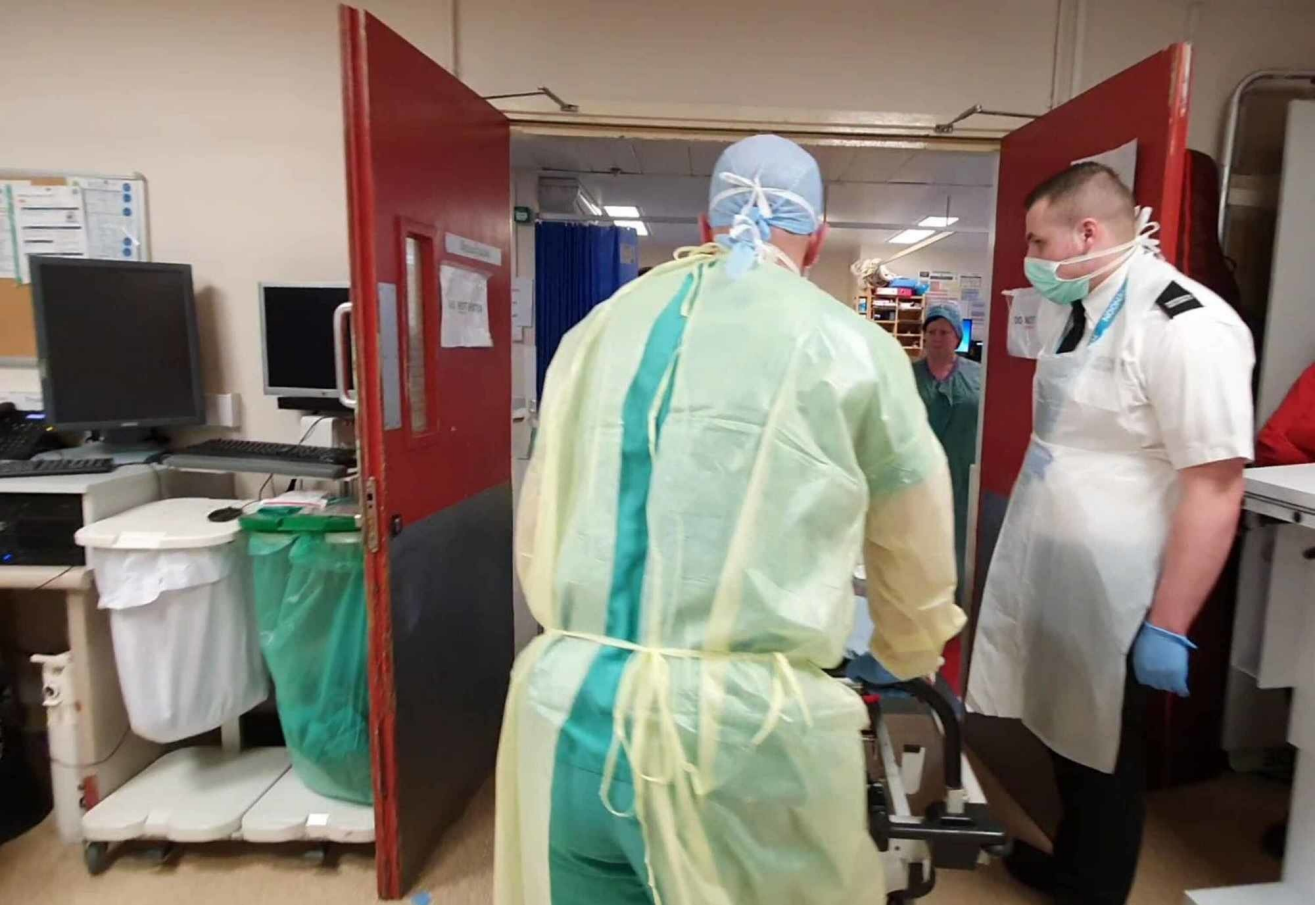
Security personnel commissioned to clear hallways of staff and patients.

1. We then included two security personnel into the Code Blue: Covid. They were commissioned to evacuate and secure the hallways and lifts leading to the intensive care unit (Figure 5).

Structure/Location

Initially, we had converted a side room that was separate but connected to the resuscitation bay as a “COVID Room”. We initially felt that this was the optimum location for such a room as it had an external entrance, separate to the main ED. This was reasonable as the logic was not to contaminate the ED making it a “confirmed/possible COVID-19” space. This however proved to be counterproductive when it involved a sick patient who required advanced airway management. The room was too small. Fitting three to four staff, a patient trolley and resuscitation equipment into this space proved to be too restrictive to effectively manage the deteriorating patient. Multiple changes were made via a protocol and structural modifications of the ED.

**Figure 6:**
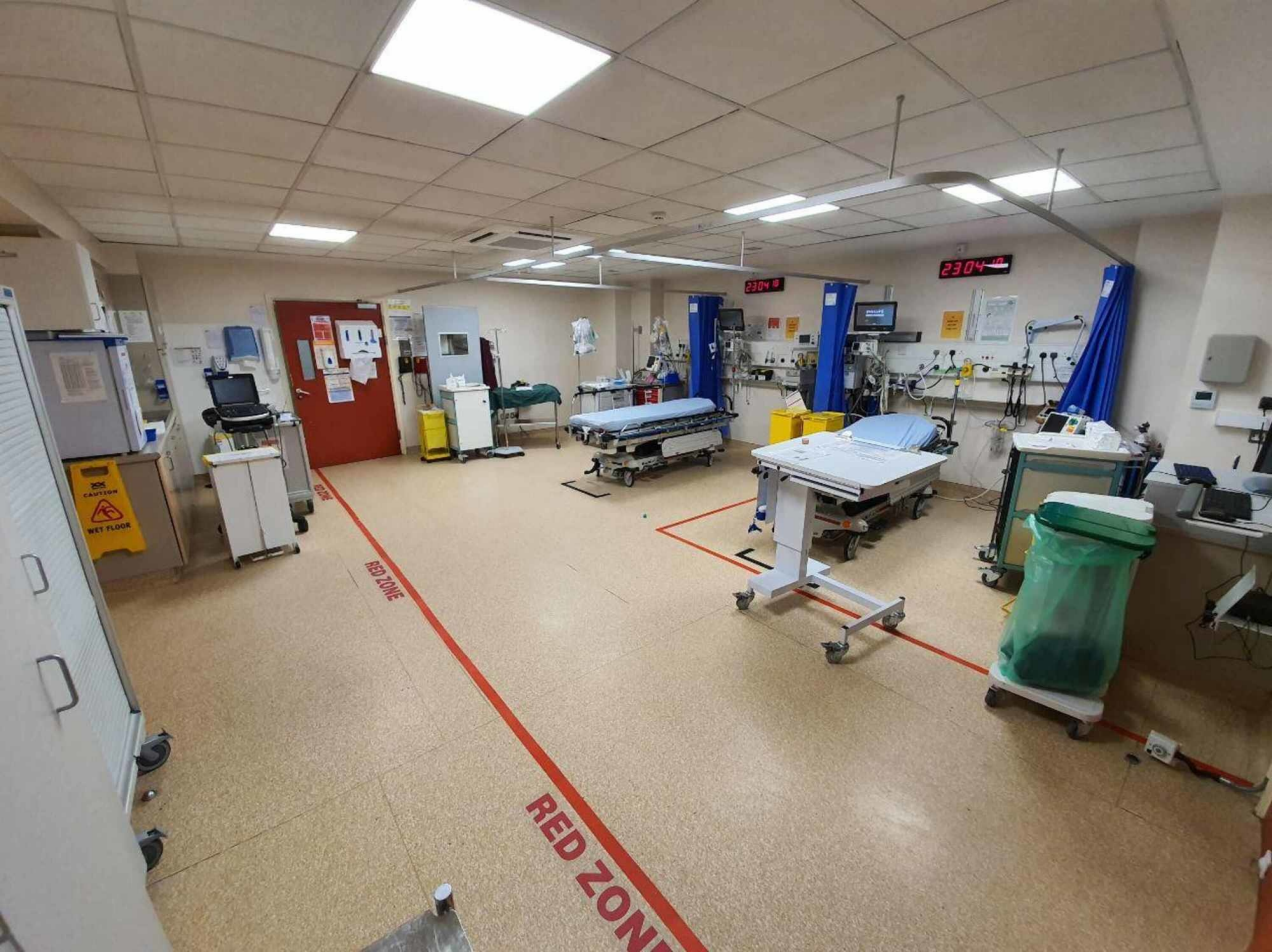
“Red” resuscitation room for potential/COVID-19 positive cases.

**Figure 7:**
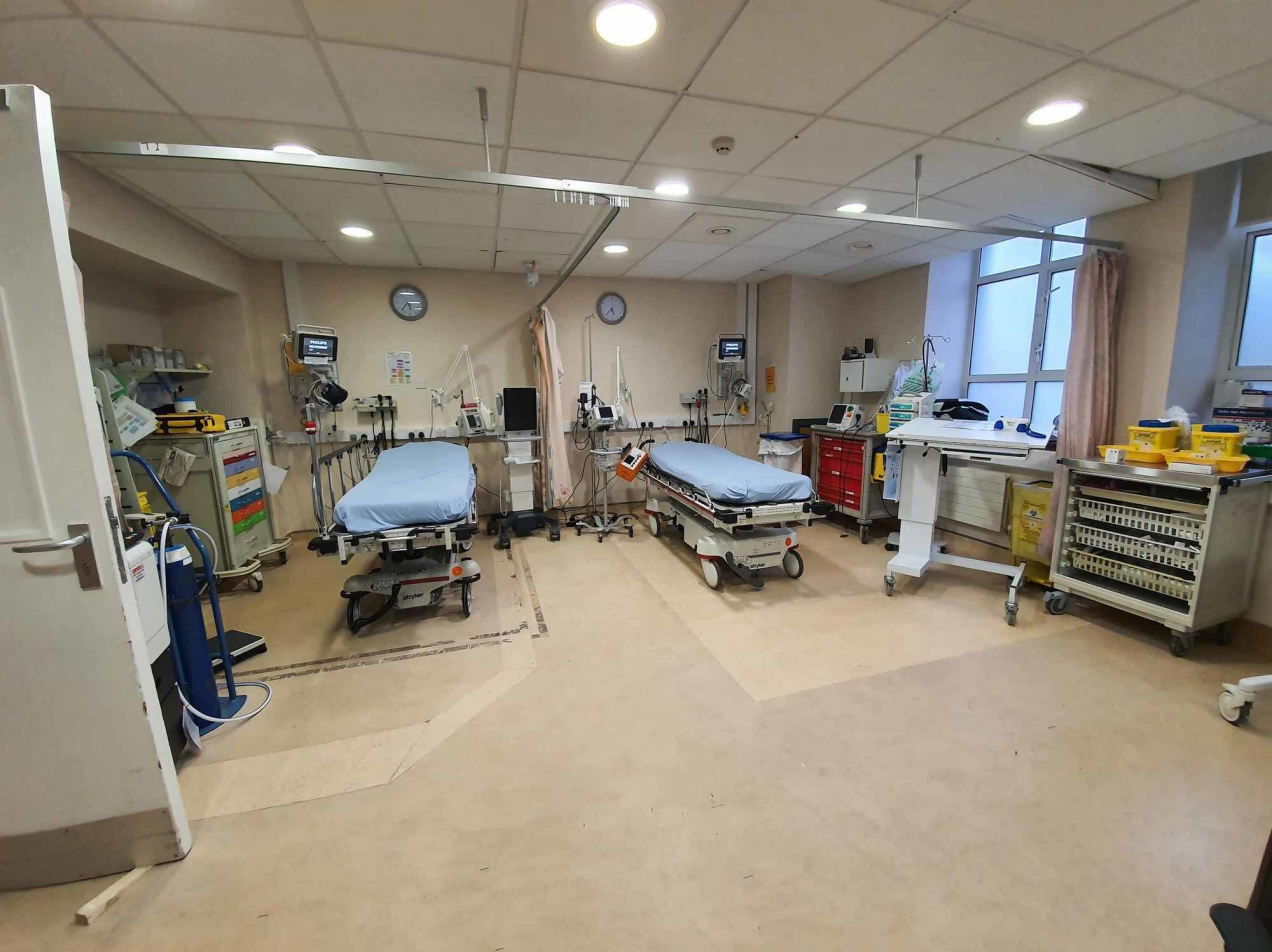
“Green” resuscitation room (converted from an ANP room originally used to treat patients with non-acute injuries). ANP: Advanced nurse practitioner

**Figure 8:**
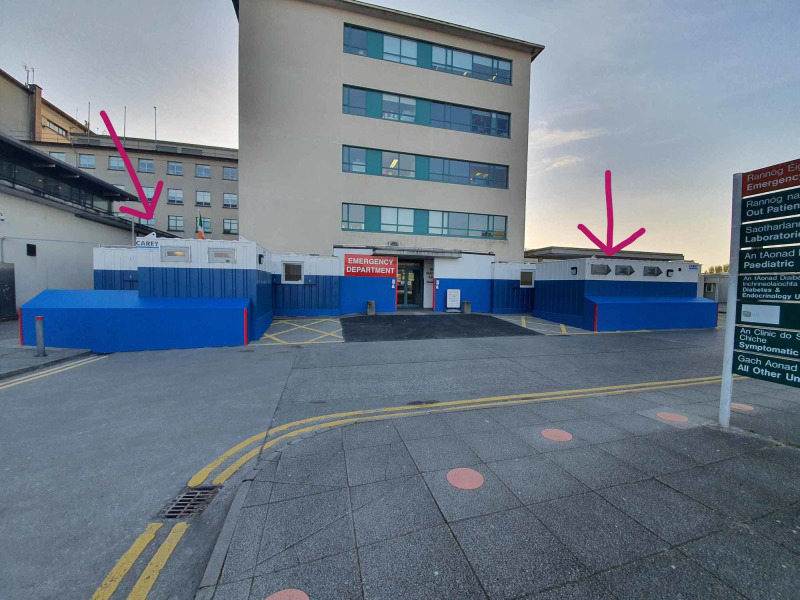
Additional extension built to function as “Red” and “Green” waiting rooms.

**Figure 9:**
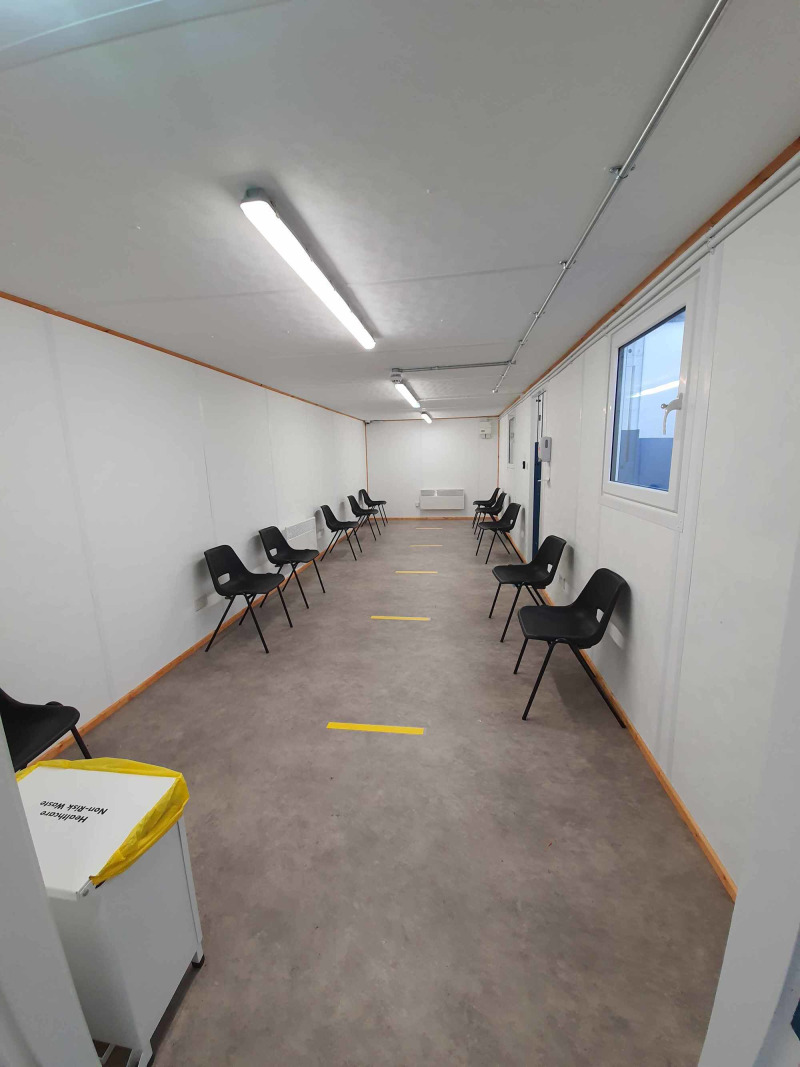
Example of the “Red” waiting room.

**Figure 10:**
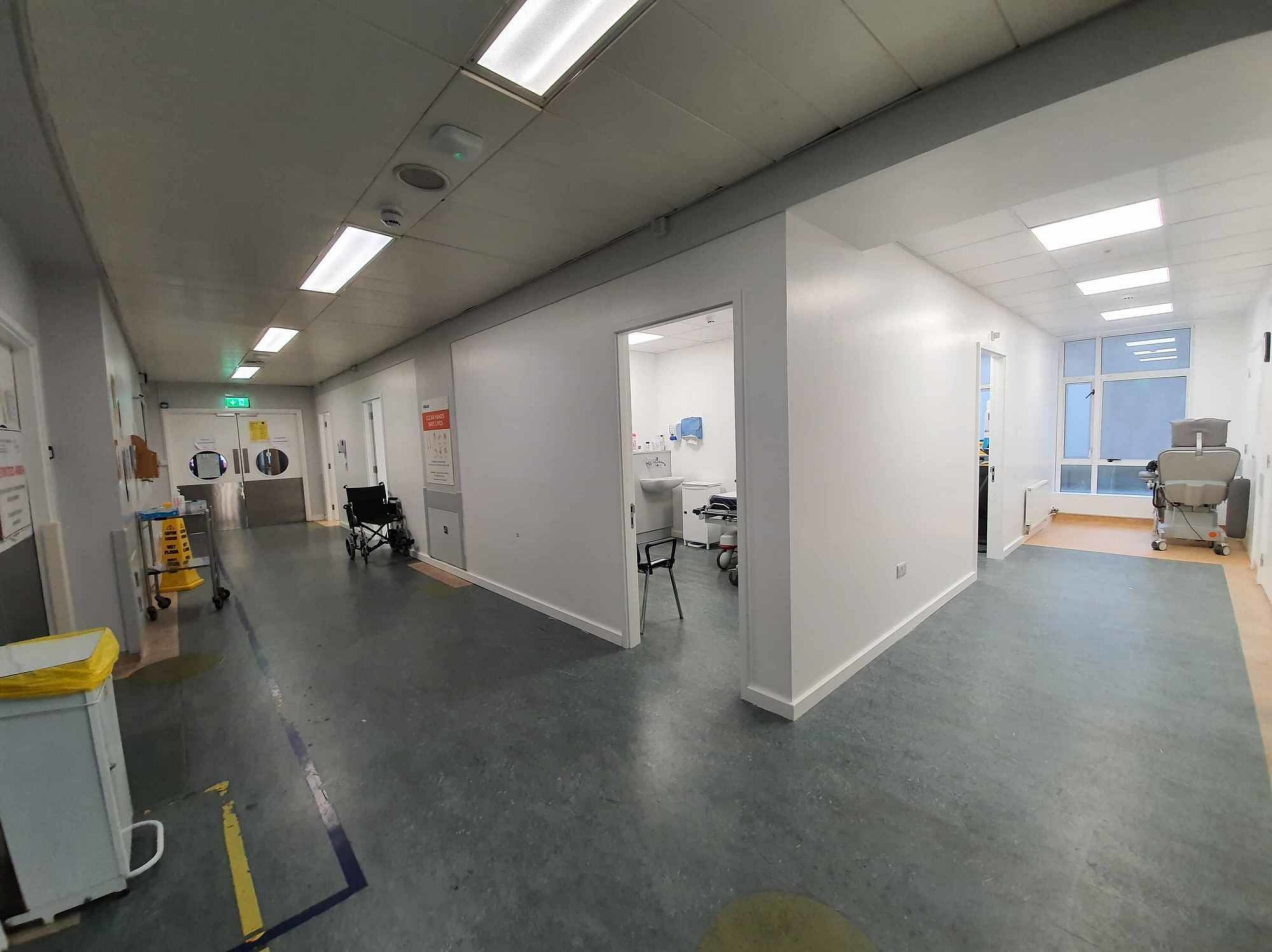
Waiting room area converted into “Red” and “Green” triage rooms.

1. A “RED” resuscitation room was established specifically for potential or positive COVID-19 cases. The resuscitation room now has markers placed on the ground specifying “red zones” to prompt clinical personnel of their respective operational zones (Figure 6).

2. The main resuscitation room was then converted for “potential/COVID-19” use. Following this, we identified a requirement of another resuscitation room. We then converted our previous “non-acute injuries” room into a separate “Green” resuscitation room. This is now used for all non-COVID patients requiring resuscitation (Figure 7).

3. We also identified the need to create separate waiting rooms for potential or confirmed COVID-19 patients, and non-COVID patients. To do this we added two extensions outside the main building of ED (Figure 8). Similar to the resuscitation rooms, these are now designated “red” and “green” waiting rooms, respectively. Each designated room is equipped with heating, ventilation, and washrooms (Figure 8). Each designated waiting room has seatings two meters apart. This was an attempt to reduce the spread of the virus while adhering to social distancing guidelines ensuring patient safety (Figure 9).

4. We applied the same principle for triage. The original waiting area was remodeled to include separate rooms for “red” and “green” triage. We saw the need to reduce the risk of cross-contamination of the potential/COVID-19 patients and non-COVID-19 patients (Figure 10).

**Figure 11:**
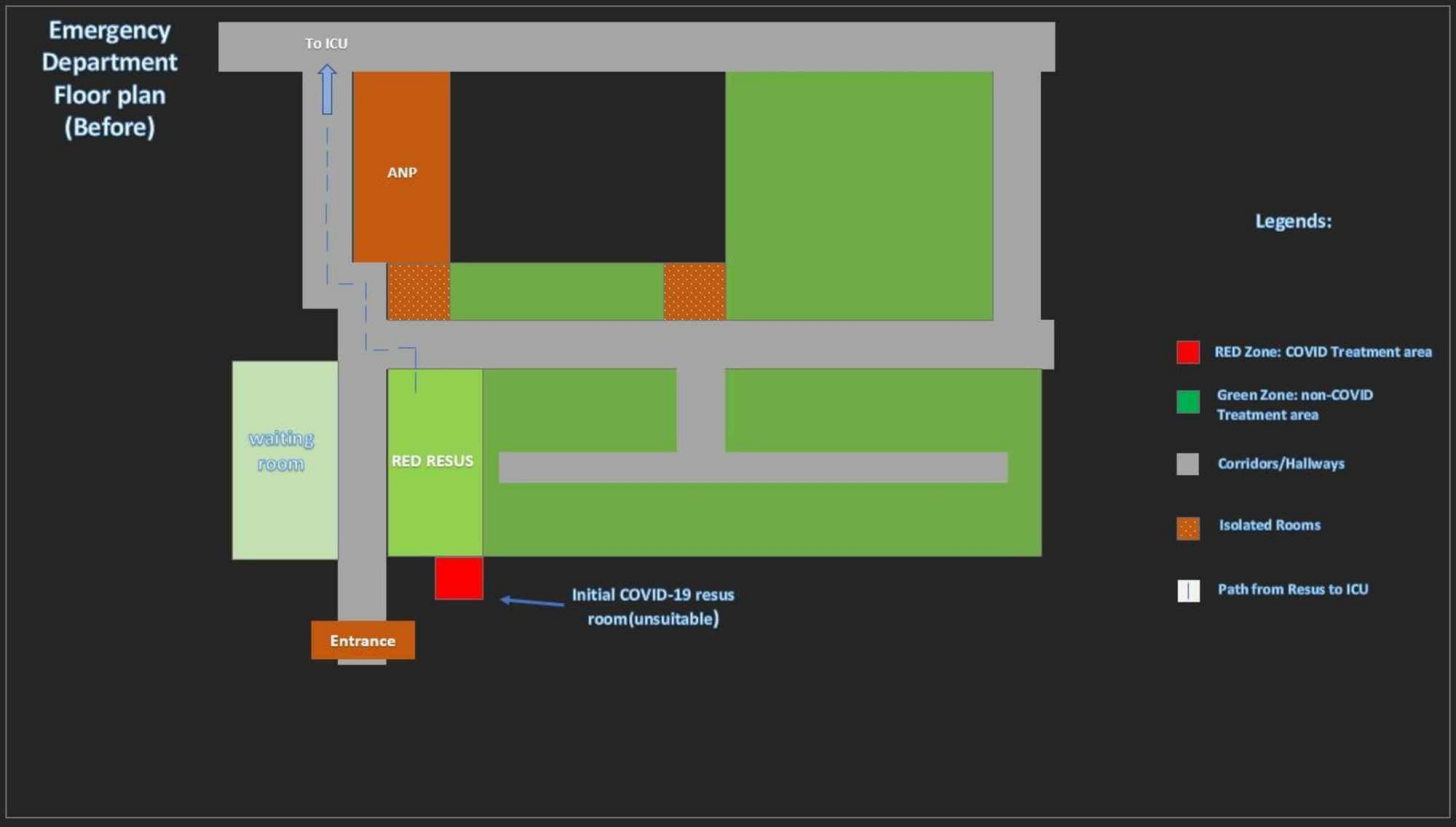
Floor plans of ED (Before changes).

**Figure 12:**
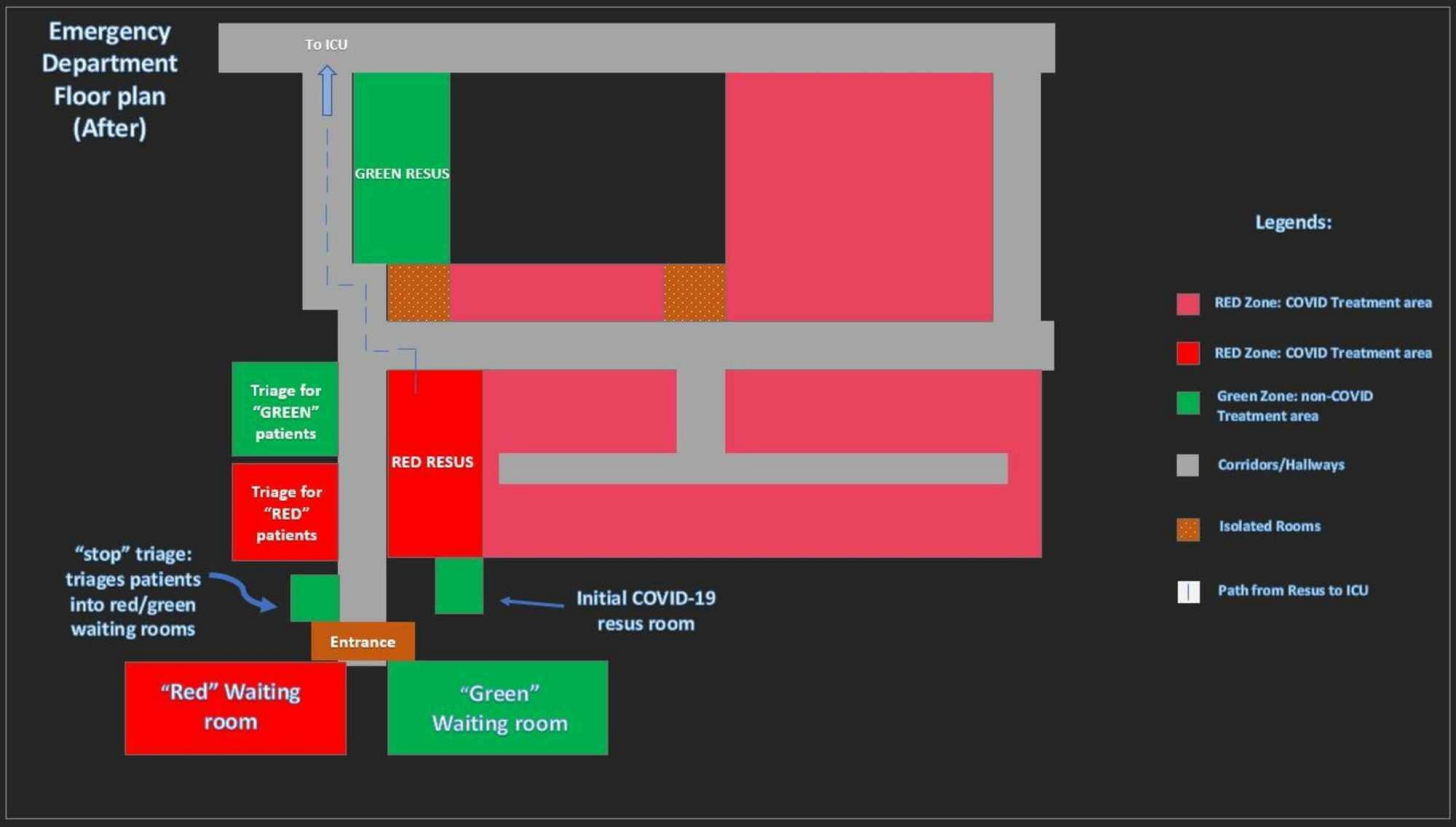
Floor plans of ED (After changes).

The overall structural changes to the emergency department are shown in Figure 11 and Figure 12.

Protocols

We introduced “CODE Blue: COVID - Anaesthetics”

1. Similar to a cardiac arrest call: when “Code: Blue” is activated, one ED registrar, one senior anaesthetic registrar, one resus nurse, and two runners are to be involved.

2. In this protocol, we corrected the RSI checklist to include a viral filter & RSI drugs in a pack.

3. A COVID-19 intubation trolley.

4. Inclusion of early anesthetics decision for intubation and escalation.

5. The number of initial participants was reduced from 10 to five (three health care professionals in the scenario and two runners).

6. On this basis, we eliminated the need for unrequired staff by administering relevant roles.

7. When ready for transfer: two security personnel would clear path and keep doors open for transfers to ICU as no porters were involved in patient transfer. The patient trolley is pushed by the anaesthetic doctor and ED nurse.

8. After the transfer, a deep clean of the room will be performed.

## Discussion

During the early phases, we decided that communication was key in our management of the outbreak.

**Figure 13:**
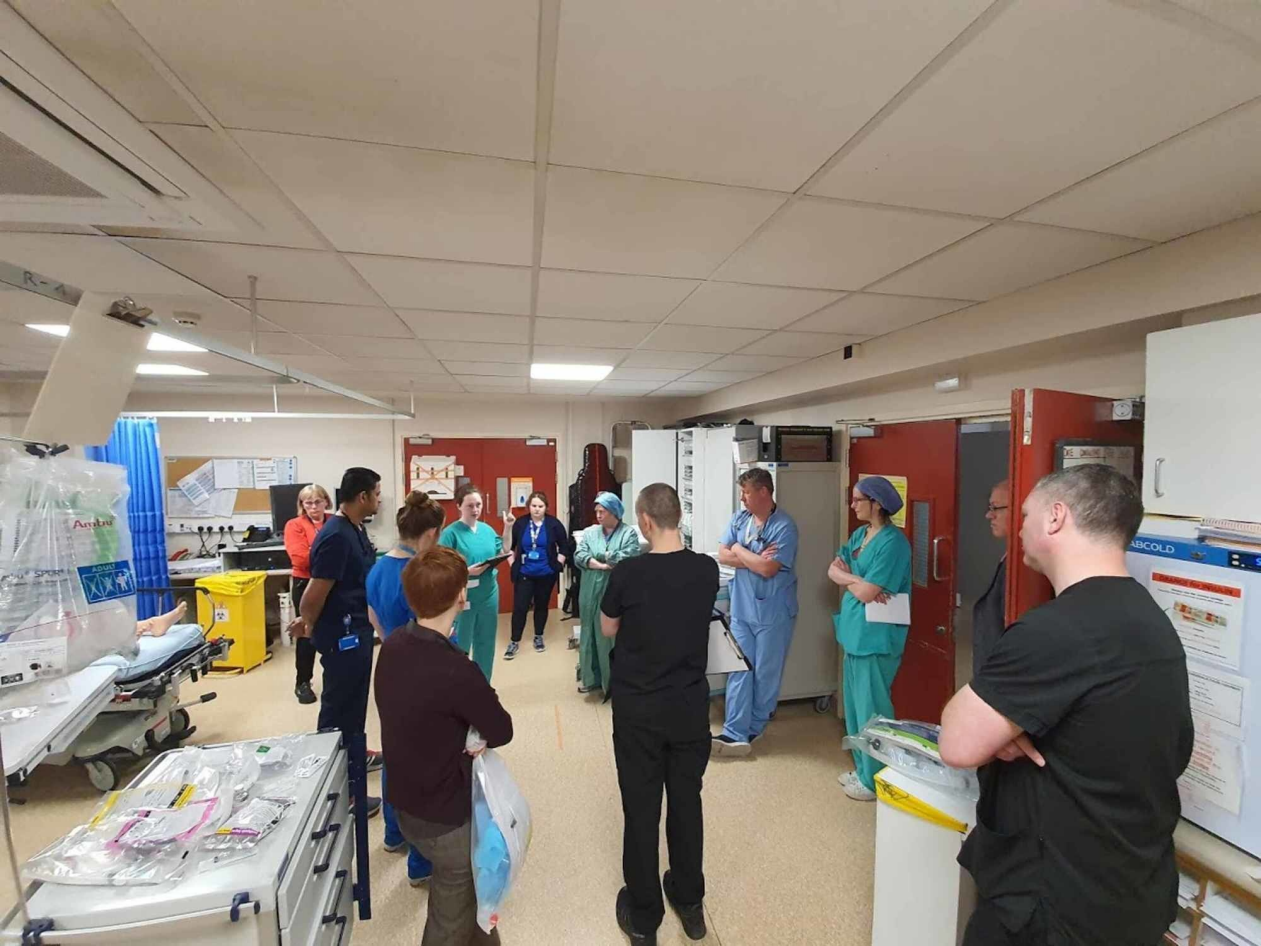
Daily meeting of senior-decision makers to update local COVID-19 guidelines and protocol.

**Figure 14:**
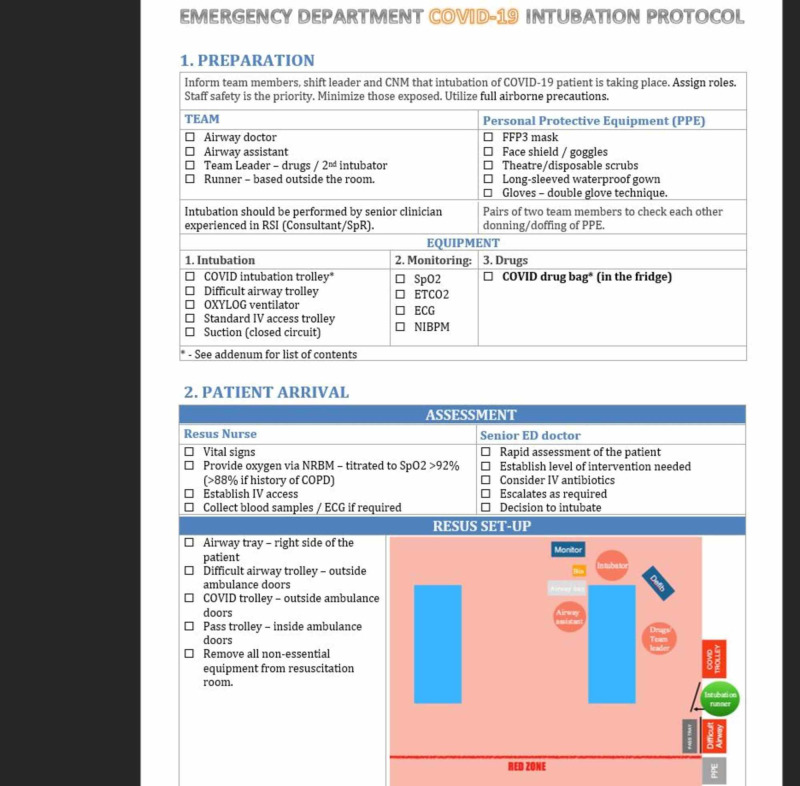
Emergency Department COVID-19 Intubation Protocol (Page 1).

**Figure 15:**
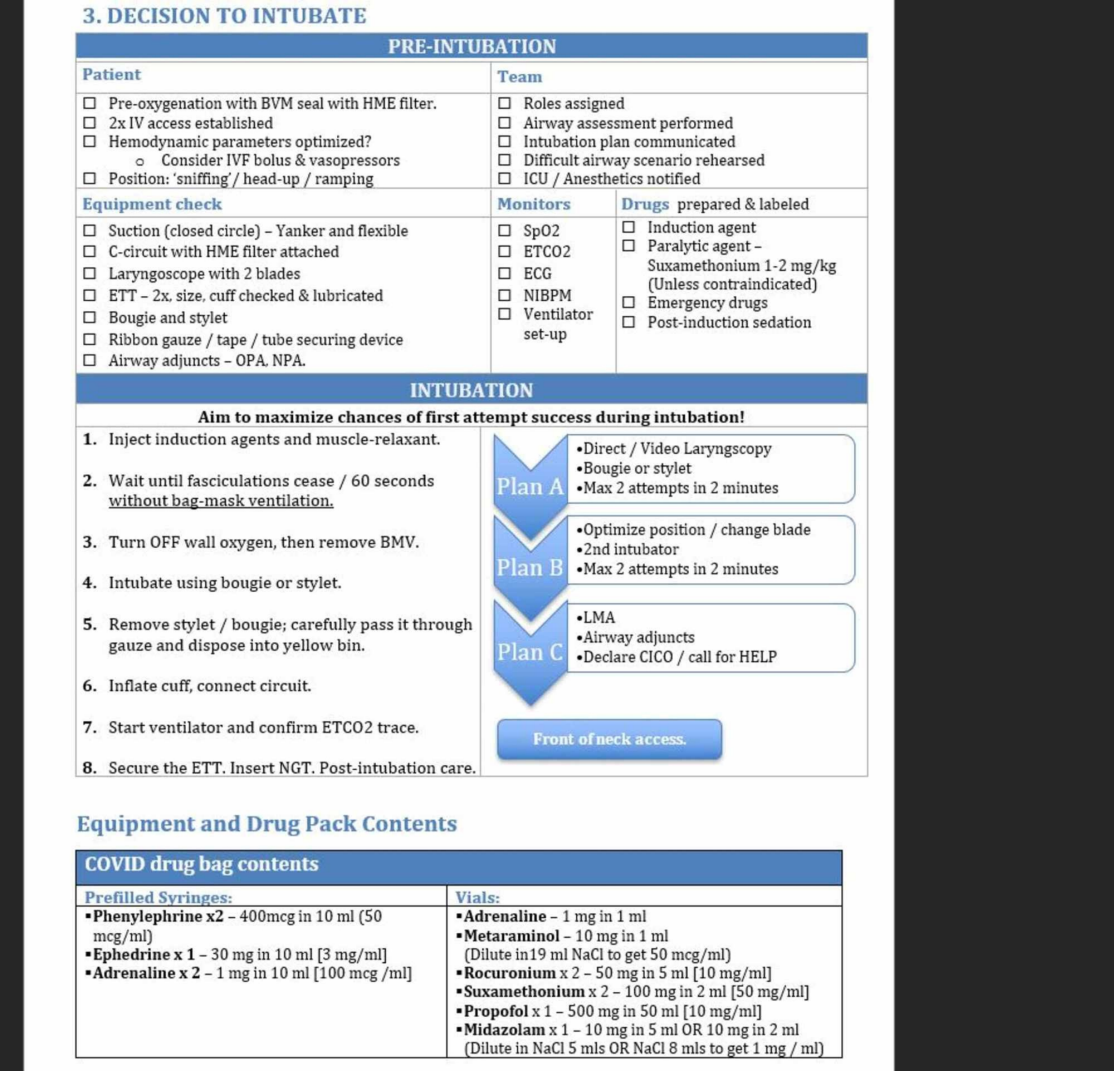
Emergency Department COVID-19 Intubation Protocol (Page 2).

Through the use of in situ simulations, hospital guidelines and protocols were modified routinely based on evolving WHO and Irish National Guidelines via daily meetings of senior decision-makers (Figure 13). During this period we introduced the two-page Emergency Department COVID-19 Intubation Protocol. Page one covers the preparation stage for intubation (Figure 14) and page two includes contingency plans with a list of equipment and drugs required (Figure 15). The updated guidelines were then emailed to all staff by the Head of HR to ensure consistency in treatment and management.

A dedicated resource folder with updates and links on COVID-19 policies and pathways for the hospital were made available through cloud and servers on every computer in the hospital. This is to ensure that there was no misleading information regarding new developments due to personnel being on shift work.

Apart from the participants and general ED & ICU staff, consultants and general managers were invited to watch. This was to demonstrate the system and structural inadequacies that were present. We found that the above personnel were paramount in this project as the structural modifications and budget allocation required approval from management.

Our project resonates with other studies looking at the use of in situ simulation to improve staff confidence level and teamwork skills. It also offers an opportunity to test the introduction of protocols, guidelines, and system changes, while observing for latent safety threats to their introduction and possible solutions [9].

## Conclusions

Due to the complex functions of every individual ED, we realized that it is not a “one size fits all” concept. However, we have demonstrated that in situ simulation is an invaluable tool in rapidly identifying system issues and safety hazards in acute care settings. Such simulations also allow for unique interdepartmental training opportunities to advocate for a common cause in these uncertain times. Therefore, conducting in situ simulations and the use of foresight, is pivotal as it could improve timely resource utilization in preparing for an outbreak. We hope that our learning points will be of value to others in preparation for this pandemic.
